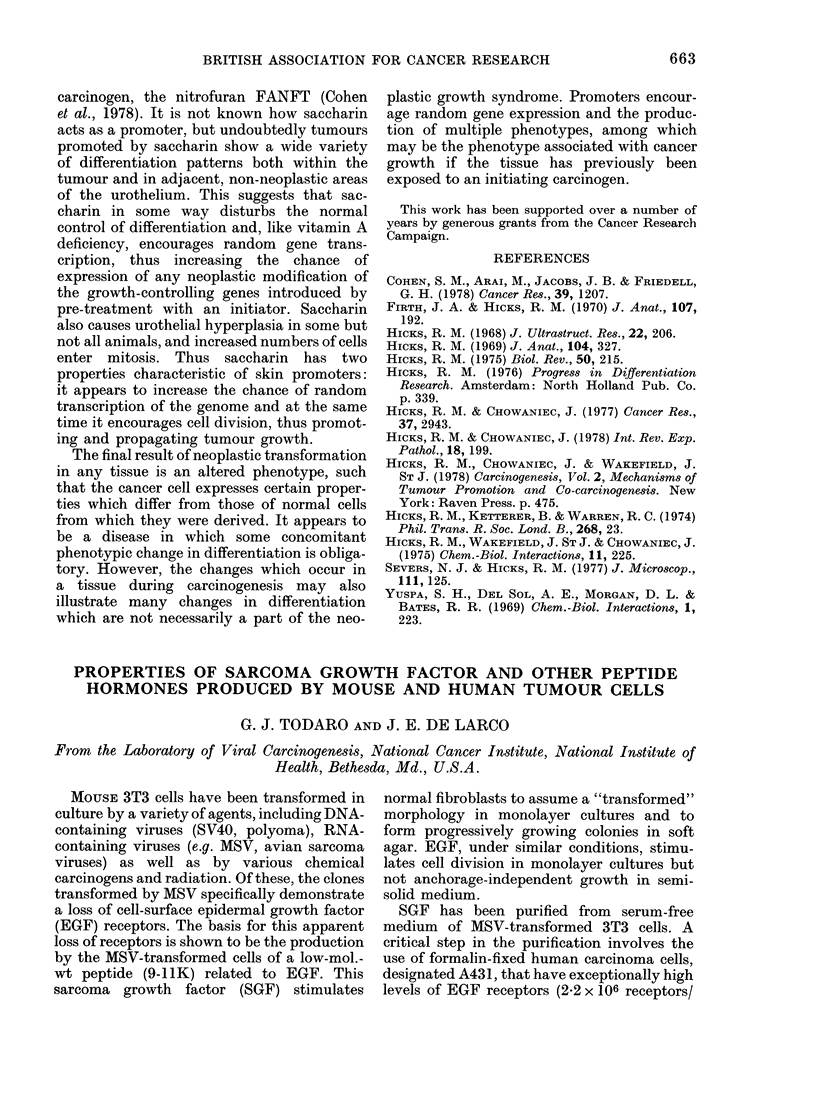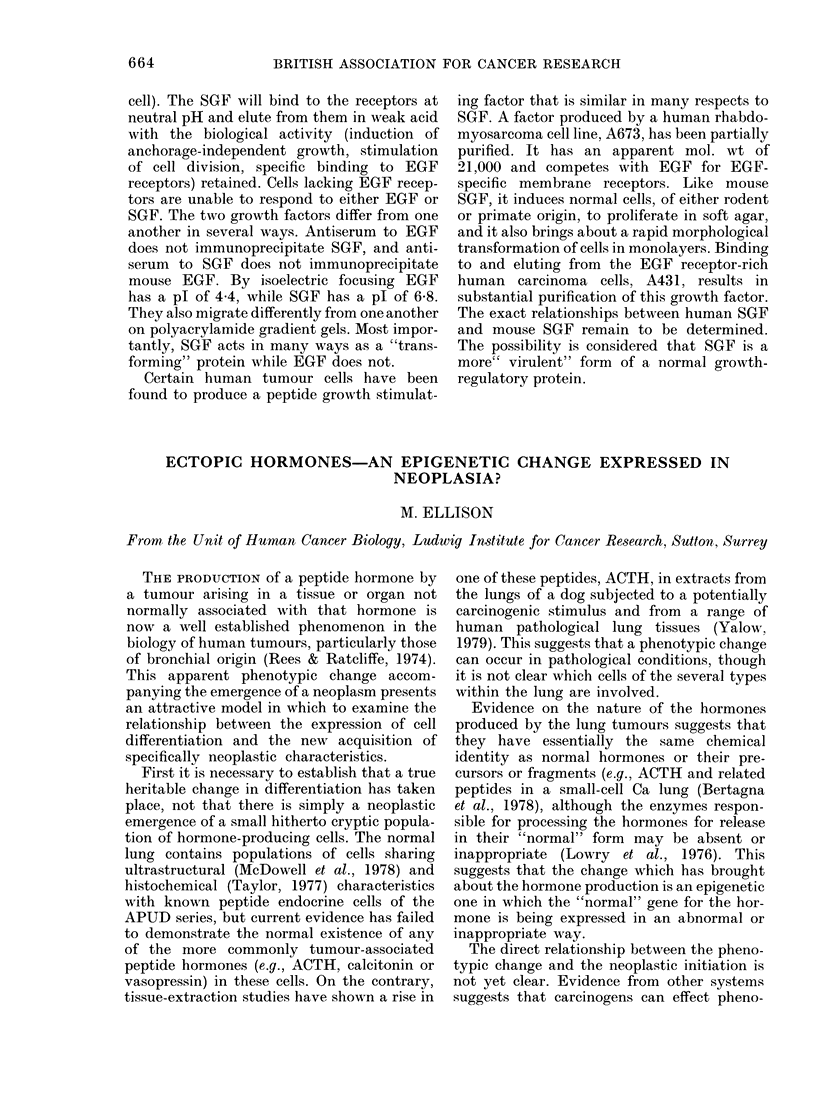# Properties of Sarcoma Growth Factor and other Peptide Hormones Produced by Mouse and Human Tumour Cells

**Published:** 1980-04

**Authors:** G. J. Todaro, J. E. de Larco


### PROPERTIES OF SARCOMA GROWTH FACTOR AND OTHER PEPTIDE HORMONES PRODUCED BY MOUSE AND HUMAN TUMOUR CELLS

G. J. TODARO AND J. E. DE LARCO

*From the Laboratory of Viral Carcinogenesis, National Cancer Institute, National Institute of Health, Bethesda, Md., U.S.A.*

MOUSE 3T3 cells have been transformed in culture by a variety of agents, including DNA-containing viruses (SV40, polyoma), RNA-containing viruses (*e.g.* MSV, avian sarcoma viruses) as well as by various chemical carcinogens and radiation. Of these, the clones transformed by MSV specifically demonstrate a loss of cell-surface epidermal growth factor (EGF) receptors. The basis for this apparent loss of receptors is shown to be the production by the MSV-transformed cells of a low-mol.-wt peptide (9-11K) related to EGF. This sarcoma growth factor (SGF) stimulates normal fibroblasts to assume a "transformed" morphology in monolayer cultures and to form progressively growing colonies in soft agar. EGF, under similar conditions, stimulates cell division in monolayer cultures but not anchorage-independent growth in semi-solid medium.

SGF has been purified from serum-free medium of MSV-transformed 3T3 cells. A critical step in the purification involves the use of formalin-fixed human carcinoma cells, designated A431, that have exceptionally high levels of EGF receptors ($2 \cdot 2 \times 10^6$ receptors/

cell). The SGF will bind to the receptors at neutral pH and elute from them in weak acid with the biological activity (induction of anchorage-independent growth, stimulation of cell division, specific binding to EGF receptors) retained. Cells lacking EGF receptors are unable to respond to either EGF or SGF. The two growth factors differ from one another in several ways. Antiserum to EGF does not immunoprecipitate SGF, and antiserum to SGF does not immunoprecipitate mouse EGF. By isoelectric focusing EGF has a pI of 4·4, while SGF has a pI of 6·8. They also migrate differently from one another on polyacrylamide gradient gels. Most importantly, SGF acts in many ways as a "transforming" protein while EGF does not.

Certain human tumour cells have been found to produce a peptide growth stimulating factor that is similar in many respects to SGF. A factor produced by a human rhabdomyosarcoma cell line, A673, has been partially purified. It has an apparent mol. wt of 21,000 and competes with EGF for EGF-specific membrane receptors. Like mouse SGF, it induces normal cells, of either rodent or primate origin, to proliferate in soft agar, and it also brings about a rapid morphological transformation of cells in monolayers. Binding to and eluting from the EGF receptor-rich human carcinoma cells, A431, results in substantial purification of this growth factor. The exact relationships between human SGF and mouse SGF remain to be determined. The possibility is considered that SGF is a more "virulent" form of a normal growth-regulatory protein.